# Exploration of the Advanced VIVO^TM^ Joint Simulator: An In-Depth Analysis of Opportunities and Limitations Demonstrated by the Artificial Knee Joint

**DOI:** 10.3390/bioengineering11020178

**Published:** 2024-02-13

**Authors:** Paul Henke, Leo Ruehrmund, Rainer Bader, Maeruan Kebbach

**Affiliations:** Biomechanics and Implant Technology Research Laboratory, Department of Orthopaedics, Rostock University Medical Center, Doberaner Straße 142, 18057 Rostock, Germany; rainer.bader@med.uni-rostock.de (R.B.); maeruan.kebbach@med.uni-rostock.de (M.K.)

**Keywords:** experimental biomechanics, joint replacement, total knee arthroplasty, joint simulator

## Abstract

In biomechanical research, advanced joint simulators such as VIVO^TM^ offer the ability to test artificial joints under realistic kinematics and load conditions. Furthermore, it promises to simplify testing with advanced control approaches and the ability to include virtual ligaments. However, the overall functionality concerning specific test setup conditions, such as the joint lubrication or control algorithm, has not been investigated in-depth so far. Therefore, the aim of this study was to analyse the basic functionality of the VIVO^TM^ joint simulator with six degrees of freedom in order to highlight its capabilities and limitations when testing a total knee endoprostheses using a passive flexion–extension movement. For this, different test setup conditions were investigated, e.g., the control method, repeatability and kinematic reproducibility, waveform frequency, lubrication, and implant embedding. The features offered by the VIVO^TM^ joint simulator are useful for testing joint endoprostheses under realistic loading scenarios. It was found that the results were highly influenced by the varying test setup conditions, although the same mechanical load case was analysed. This study highlights the difficulties encountered when using six degrees of freedom joint simulators, contributes to their understanding, and supports users of advanced joint simulators through functional and tribological analysis of joint endoprostheses.

## 1. Introduction

In the human body, joints must withstand high mechanical stresses [1] during the lifetime. Pathological changes, like degenerative joint diseases, can impair their functionality. If conservative therapy options have been exhausted, the implantation of joint endoprostheses remains a last resort to restore joint function. The development of joint endoprostheses requires extensive preclinical investigations. Biomechanical investigations can be divided into wear tests as well as kinematic and dynamic movement analyses. The wear tests vary in complexity, from simple pin-on-disk tests to investigating material combinations and lubrication media [2] to six-dimensional movements, such as those analysed in total hip and knee implants, according to ISO 14242/43 [3,4], using wear simulators. To investigate the various implant design parameters, kinematic and dynamic analyses are often performed using customised test devices or robot setups. However, standard wear tests do not always correspond to the physiological boundary conditions [5] and are only partially able to characterise the behaviour of individual implant designs [6]. On the other hand, custom test rigs are usually limited to single joints, whereas the human body has more than 100 real joints. Furthermore, the use of robots in research can be complex to handle and time consuming [7].

Contrarily, computational simulations are efficient to analyse the effect of various test parameters on joint dynamics and can support clinical decision making [8,9,10,11,12]. However, it remains challenging to effectively replicate realistic contact conditions [13] which therefore need to be validated experimentally [14].

A promising solution to fill this remaining gap is advanced joint simulators, such as the VIVO^TM^ (Advanced Mechanical Technology, Inc., Watertown, MA, USA), which promises a combination of adaptive design to test different joints and programming simplicity. The VIVO^TM^ joint simulator is capable of controlling six degrees of freedom (DOF), either kinematically or dynamically (hybrid force-position control). Furthermore, it has the possibility to consider up to 100 virtual ligaments during the examinations by virtually applying forces and moments during the movement, depending on the joint pose [15]. These features provide the possibility to use the VIVO^TM^ joint simulator for a variety of joints, for example the elbow [16,17], patellofemoral joint [18,19], tibiofemoral joint [19,20,21,22,23], and mandible joint [24].

However, the sensitivity of the overall functionalities of these advanced simulators to certain test setup conditions, such as joint lubrication or control algorithm, has not been thoroughly examined yet. Additionally, some of the test-setup conditions are relevant for general experimental testing. These include, for example, lubrication, for which many different approaches are known in the literature [3,4,14,21,25,26,27,28,29,30]. In experimental investigations of joint endoprostheses, additional lubrication is neglected due to the self-lubricating properties of polyethylene, e.g., studies with test rigs [25], robots [14], and wear simulators [26]. Other studies used silicone oil to enforce ideal lubrication behaviour [21].

Therefore, the aim of this study is to analyse the fundamental functionality of the VIVO^TM^ joint simulator for biomechanical testing of total knee endoprostheses. For this purpose, using the example of a passive flexion–extension knee movement, the influence of different test parameters, e.g., control methods, implant embedding, testing speed and lubrication, is analysed. Furthermore, repeatability and kinematic reproducibility are evaluated by comparing a purely kinematic load case with its dynamic equivalent, regarding the imposed joint reaction forces. Thus, this study aims to highlight the capabilities and limitations of the advanced VIVO^TM^ joint simulator and to point out its advantages compared to previous types of joint simulators and its sensitivities concerning specific test setups.

## 2. Materials and Methods

### 2.1. Six Degrees of Freedom Joint Simulator VIVO^TM^

#### 2.1.1. Physical Setup

**Figure 1:**
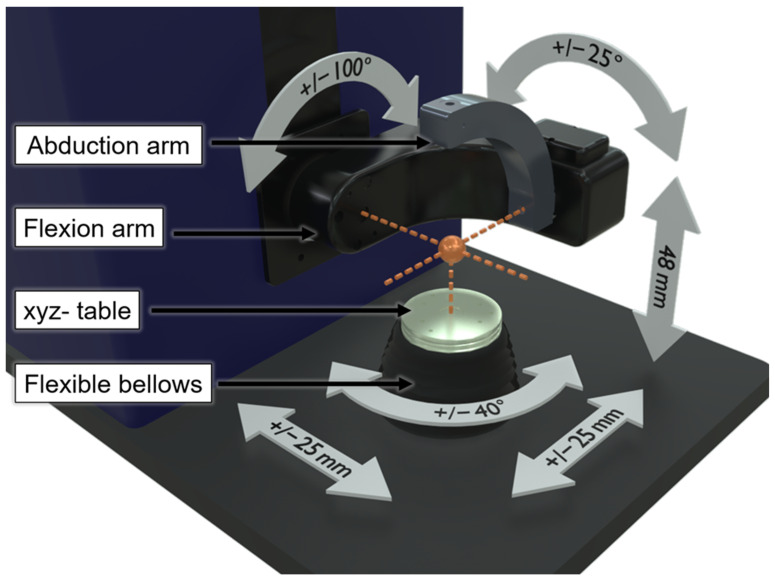
Physical setup of the VIVO^TM^ joint simulator with the respective components and illustration of the degrees of freedom as well as the maximum range of motion of the actuators.

The moving parts of the VIVO^TM^ joint simulator consist of one lower and one upper actuator, whereas the upper actuator can be divided into a flexion and abduction arm, thus enabling two rotations. The lower actuator, the xyz table can perform three translations and one rotation. A 6-DOF load cell underneath the xyz table measures all forces and moments applied to the joint and is protected from lubricants and impurities by flexible bellows. The moving parts of the VIVO^TM^ joint simulator and the corresponding DOF are visualised in Figure 1.

#### 2.1.2. Definition of Coordinate Systems

**Figure 2:**
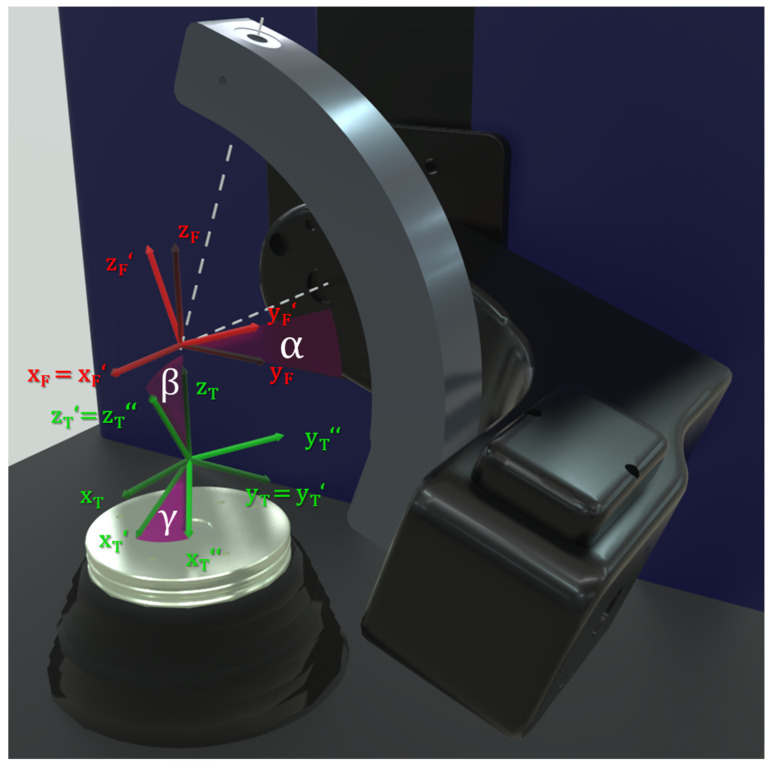
The coordinate system “F” of the upper joint partner (red) is centred in the intersection of the flexion and abduction arm axes and can only be rotated about its *x*-axis (angle α). The position of the lower joint partner’s coordinate system “T” (green) is not further defined and can be freely chosen, as can the rotation about the y- and z-axes (angle β and γ).

The VIVO^TM^ joint simulator can be used to analyse a broad range of joints, for example the elbow [16,17], patellofemoral joint [18,19], tibiofemoral joint [19,20,21,22,23], and mandible [24] joint. The VIVO^TM^ joint simulator disposes of two different control modes—the “Cartesian” mode, which interprets the kinematics according to the actuator axes (Figure 1), and the “Grood & Suntay (GS) mode”. As the GS mode is the mode in which each DOF can be force and position controlled, and in which virtual ligaments can be applied according to Wismans and Blankevoort [31,32], it will be focused on in our present study. The GS mode interprets input and output kinematics according to the GS coordinate system [33], which are the lateral–medial, anterior–posterior, and inferior–superior translations as well as the flexion–extension, the abduction–adduction and the external–internal rotation. Prior to testing, a suitable positioning of the implant-bearing coordinate system needs to be considered and defined. The definitions of the coordinate systems for the upper and lower joint partner are not entirely freely selectable but are predetermined by certain DOF. It is clearly defined that the position of the coordinate system “F” of the joint partner connected to the upper actuator is fixed (at the intersection of the abduction arm axis with the flexion arm axis) and can only be rotated around its *X*-axis. Contrary, the coordinate system “T” of the joint partner connected to the lower actuator cannot be rotated around its *x*-axis but can be manipulated in its remaining DOF (Figure 2). Before determining the coordinate systems, the actuators need to be moved to a known pose, the “Reference Pose”. For this task, the manufacturer recommends a hybrid force- and position-controlled actuation.

#### 2.1.3. Force and Moment Interpretation

**Figure 3:**
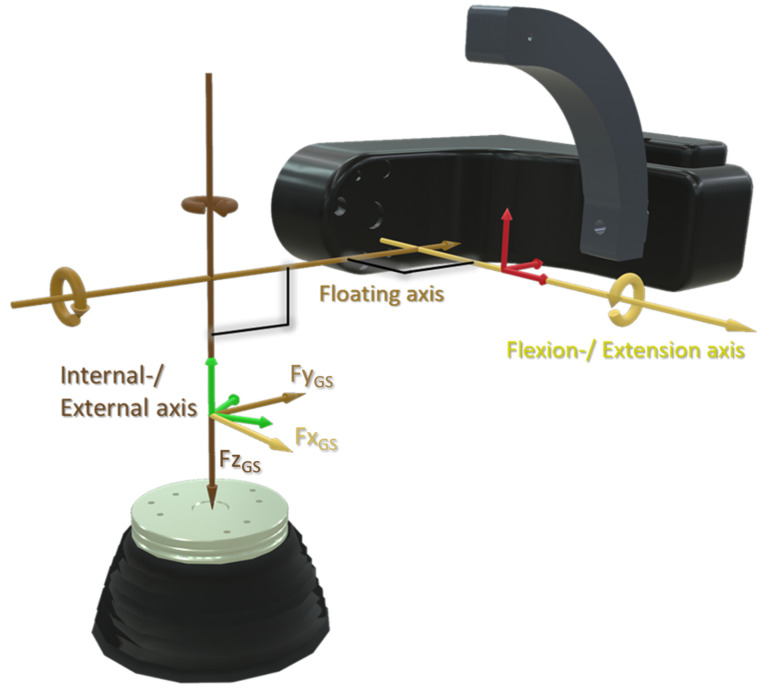
Force and moment directions Fx_GS_, Fy_GS_ and Fz_GS_, interpreted by the VIVO^TM^ joint simulator in “Grood and Suntay” (GS) mode are obtained by parallel shifting the GS axes [15] onto the lower actuator coordinate system “T”.

After transferring all six parameters required to define the coordinate systems, the movements are interpreted in GS coordinates, which therefore generally do not coincide with the actuator axes (Figure 1). The joint forces and moments are output relative to the lower coordinate system. To obtain the force directions, the three axes described in the GS coordinate system are shifted parallel to the origin of the lower actuator coordinate system (T), which is visualised in Figure 3.

The VIVO^TM^ joint simulator offers the possibility to virtually implement ligaments as non-linear force elements, according to Wismans and Blankevoort [31,32]. In the reference pose, the user can define the mechanical properties of ligament parameters of up to 100 ligaments. For each ligament, this includes the ligament attachment points, the ligament stiffness, and the reference strain (strains of the ligaments in the reference pose). Depending on the pose of the upper and lower coordinate systems, forces are calculated for each ligament, which act from attachment point to insertion point.

#### 2.1.4. Controller Design

Loads and kinematics are transferred via waveforms to the VIVO^TM^ joint simulator. Additionally, to basic waveforms like sine, sawtooth, triangle, and square, it also allows for arbitrary waveforms to be imported via .csv or .txt files. As the VIVO^TM^ joint simulator is designed for periodic load cases, e.g., human movement scenarios, the starting and end point of each waveform will be aligned for continuous cyclical testing. Because of the continuous cycling testing, the VIVO^TM^ joint simulator can feature an “iterative learning control” (ILC) algorithm to minimise the errors through testing or can be controlled traditionally via proportional–integral (PI) control. The ILC records the tracking error over one or more waveform cycles, analyses the data, and adapts the control to improve the tracking. This process is repeated continuously and can reduce the tracking error to insignificance [15].

### 2.2. Experimental Configuration for Testing of Total Knee Endoprostheses

To analyse total knee endoprostheses, a bicondylar cruciate-retaining knee endoprosthesis (P.F.C.^®^ Sigma, DePuy Synthes, Raynham, MA, USA) with a femoral component, a tibial insert and a tibial component were used. A virtual ligament apparatus for the tibiofemoral joint from previous simulation studies was transferred to the joint simulator [34]. The ligament apparatus consisted of 13 different force elements whose stiffnesses and reference strains are shown in Table 1. More precisely, the following tibiofemoral ligament structures were included: the anterior and posterior bundle of the posterior cruciate ligament (aPCL/pPCL), the anterior medial collateral ligament (aMCL), posterior medial collateral ligament (pMCL), distal medial collateral ligament (dMCL), anterior and posterior lateral collateral ligament (aLCL/pLCL), proximal and distal oblique popliteal ligament (pOPL/dOPL), arcuate popliteal ligament (APL), lateral posterior capsule (lpCAP) and the medial posterior capsule (mpCAP). The anterior cruciate ligament was not considered.

**Figure 4:**
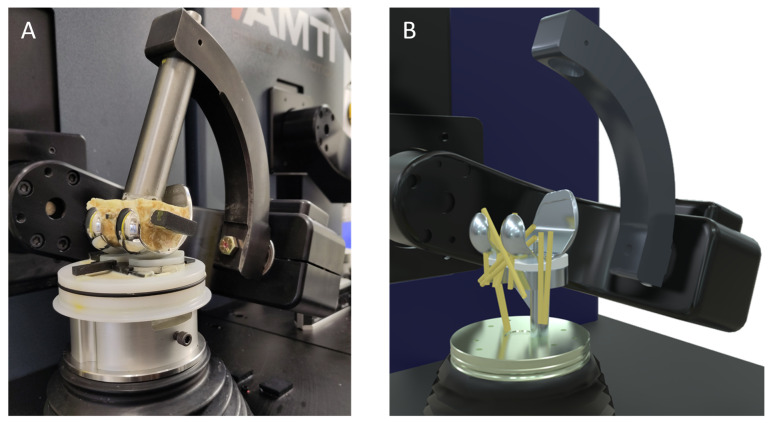
Experimental test setup with the femoral component attached to the upper and the tibial tray attached to the lower actuator with self-curing polymer (**A**). Moreover, the virtually implemented ligaments are depicted (**B**).

A custom-made steel adapter was used to attach the femoral component to the upper actuator, while the manufacturer-supplied mounting plate was used to embed the tibial tray in the lower actuator. To ensure the correct positioning, additively manufactured parts were produced using the 3D printer Prusa MK3S+ (Prusa Research, Prag, Czech Republic), in order to position the implant components exactly during the embedding in self-curing polymer (Rencast^®^ FC 52/53 Isocyanat and FC 53 Polyol, Huntsman Advanced Materials GmbH, Bergkamen, Germany). The experimental test setup and the virtually applied ligaments are visualised in Figure 4.

#### Functional Principle

In this study, the performance of the VIVO^TM^ joint simulator was investigated under different influencing parameters during passive flexion–extension movement of the knee. For this load case, the flexion–extension movement was position-controlled from 0° to 100° to 0° as a sine wave. All three translational movements (lateral–medial, anterior–posterior, inferior–superior) and the two remaining rotational movements (abduction–adduction, external–internal rotation) were force controlled to zero. With the joint reaction forces and moments set to zero, the total knee implant should find its equilibrium during flexion, with the joint contact and ligament forces balancing each other.

### 2.3. Parameter Variations for Sensitivity Analysis

The focus of this study was the sensitivity analysis of the passive flexion–extension movement under varying test parameters. Therefore, to classify the impact of the different test parameters an initial load case for comparison was needed and referenced as “reference test”. The “reference test” was a passive flexion–extension movement controlled with the ILC at 0.25 Hz frequency, resulting in a duration of a single cycle of 4 s, and without lubrication of the implant components. The parameters control method, repeatability as well as kinematic reproducibility, the effect of the flexible bellows, waveform frequency, the influence of lubrication, and implant embedding were analysed.

#### 2.3.1. Influence of Control Method

As mentioned above, the VIVO^TM^ joint simulator features two different control modes—ILC and PI. The ILC uses haptic mapping, an automated process prior to the actual test by which the machine develops an initial compensation profile for a more precise tracking of the waveform templates [15]. To evaluate the differences between haptic mapping and ILC, the inferior–superior force tracking error of the haptic mapping was compared with the second cycle after the completed haptic mapping. The first cycle was excluded from evaluation due to the transition time. According to the manufacturer, the ILC updates the compensation profile every third cycle to reduce the tracking error within more and more cycles. To compare ILC and PI control, the resulting tracking error during the passive flexion were compared using the root mean square error (RMSE) of all six DOF and the development of the flexion–extension angle over 20 cycles. Additionally, the ILC was compared to classic PI control after 49 cycles, with respect to the inferior–superior force tracking error. Of particular interest was to what extent the ILC is able to minimise the tracking errors over the cycles for two sets of control parameters. The control parameters are fully listed in Table A1 and Table A2 in the Appendix A. Table A1 lists the control parameters that are used to capture the “reference test” whereas Table A2 lists the control parameters that are referred to as adjusted parameters in this article and feature a proportional-controller (P-controller) of the inferior–superior DOF, increased by factor four as well as the maximum rate increased by 50% compared to the control parameters of the “reference test”.

#### 2.3.2. Repeatability and Kinematic Reproducibility

Within this study, both the repeatability and kinematic reproducibility were examined. For repeatability, the “reference test” was repeated a second time several days after the initial test. In this process, the reference pose was also readjusted. Further, to verify the kinematic reproducibility of the joint simulator, the passive flexion was imposed purely kinematically. In this scenario, all six DOF were actuated in position control. For this purpose, the kinematics of the five cycles (296 to 300) of the “reference test” had been averaged and then transferred to the simulator as input values. To evaluate the performance of the ILC for a purely kinematic test, and to verify how accurately the simulator can replicate kinematics control wise, the RMSE of the tracking error was calculated. Furthermore, examining the correlation between the curves of contact force and moment with the “reference test” should provide insights into the repeatability and kinematic reproducibility.

#### 2.3.3. Effect of the Flexible Bellows

**Figure 5:**
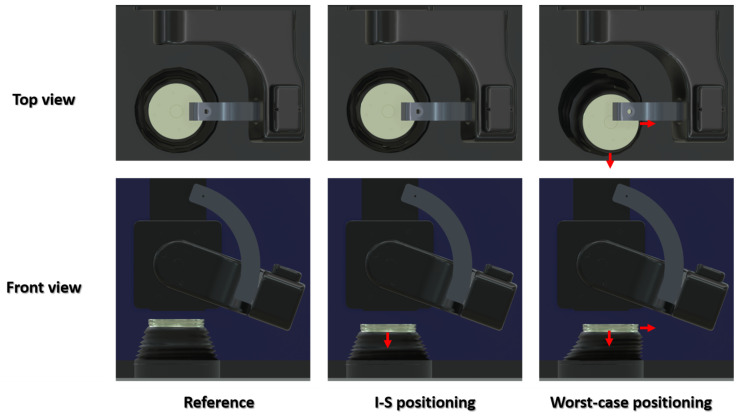
Before testing, the force transducer was set to zero in three different lower actuator positions. For the “I-S” positioning the lower actuator was moved downwards relative to the “reference”. In the “worst-case” position, the lower actuator was also shifted into lateral–medial and anterior–posterior direction.

Previous internal investigations with the VIVO^TM^ joint simulator led to the assumption that the flexible bellows could possibly lead to distortions in the test results. As the forces and moments are measured using a 6-DOF load cell underneath the xyz table, any tensile forces from the bellows are also included in the measurement. The attachment of the flexible bellows to the lower part of the xyz table can result in forces that act relatively far away from the coordinate system of the lower actuator. As a result, small forces may lead to distinct moments. For this reason, the previous purely kinematic load case was repeated, this time excluding any implants. Thus, there was no contact between the two sliding partners, and a virtual ligament apparatus was also not considered. Unlike all of the other tests in this study, the bellows used to protect the simulator from lubricants and impurities were installed as intended by the manufacturer. The forces and moments measured during this kinematic load case were almost exclusively due to the tensile forces of the flexible bellows, as there are no contact nor ligament forces (inertia forces of the lower actuator are considered by the simulator). Before each test, the load cell was manually set to zero. In this case, two positions had to be chosen where the flexible bellows were already tensioned (Figure 5). When set to zero, the implant components must not be in contact as it would distort the calibration, making the reference pose unsuitable for most cases. In the first chosen position, referred to as inferior–superior (“I-S”) positioning, the lower actuator was moved 10 mm downward before it was set to zero. In the second position, the “worst-case” positioning, the table was also moved into a maximum medial and anterior deflection before set to zero. The previously recorded, purely kinematic movement of the lower actuator during passive flexion–extension movement was then imposed. In the present study, the effects of the bellows on the contact forces and moments for two axes measured by the load cell were analysed.

#### 2.3.4. Effect of the Waveform Frequency

The effect of the waveform frequency on the resulting dynamics was investigated. With respect to the joint simulator, the impact of a higher simulator speed on the tracking error should be investigated. Therefore, the passive flexion–extension movement was controlled with a waveform frequency of 0.1 Hz and 0.5 Hz, additionally to the 0.25 Hz of the “reference test”. For the passive flexion, this equated to mean flexion angular velocities of 20, 50, and 100°/s and the corresponding maximum angular velocities of approx. 39, 78, and 157°/s. For comparison, the average measured velocity of the STAN data set of the CAMS project [35] for level walking is approx. 77°/s with a maximum angular velocity of approx. 239°/s, while the ISO 14243 [4] even reaches values of approx. 138°/s and 325°/s, respectively. To evaluate the effect of the waveform frequency, the inferior–superior contact force and the flexion–extension contact moment, as well as the RMSE of the tracking error of all six DOF, were investigated.

#### 2.3.5. Effect of Lubrication

The influence of the lubrication on the joint simulator performance was analysed with respect to the metal-on-polyethylene bearing of the total knee endoprosthesis tested. Polyethylene is an effective self-lubricating material and is often tested without additional lubrication [25] as well as with silicon oil to enforce ideal lubrication [21]. In contrast to the dry “reference test”, the passive flexion–extension test was repeated with silicon oil (Silikonöl Typ 350, Caesar & Loretz GmbH, Hilden, Germany) as the lubricant to compare the two types of lubrication. To evaluate the effect of the lubrication on the anterior–posterior and inferior–superior contact forces as well as the flexion–extension and external–internal rotation contact moments were visualised. Additionally, the RMSE of the tracking error of all six DOF were calculated.

#### 2.3.6. Sensitivity to Embedding of the Implant Components

In the next step, the influence of the embedding of implant components was analysed by simulating varying reference poses. To assign coordinate systems for the upper and lower actuators, it is necessary to first approach a known reference pose, as already described. Embedding is always subject to a certain error. Hence, the ISO 14243 standard [4], for example, grants a tolerance of 1° for embedding the tibial vertical axis. Since the implemented ligament apparatus, particularly the reference strain, is based on the reference pose, an embedding has a direct influence on the resulting ligament forces. To quantify this influence exemplary, varying embedding was simulated by manipulating the reference pose. Specifically, an artificial malalignment was analysed by simulating a ±1° adduction deviation of the femoral component and a ±0.5 mm inferior–superior shift of the tibial component. This was achieved by adjusting the position of the implants before setting the reference pose.

It should be noted that changing the tibial coordinate system also changes the axes in which the respective forces and moments are analysed. For example, the superior–inferior shift changes the lever arm between the tibiofemoral contact force and the evaluating coordinate system, resulting in different measured moments. However, the changes in the context of these investigations were so small that possible changes in the dynamics could be expected solely due to the different pretension of the virtual ligament apparatus.

By adducting the femoral component before setting the reference pose, the flexion axis was also inclined by ±1°. However, for such small angles, the impact of implant embedding on the resulting joint kinematics was negligible.

To evaluate the effect of the embedding of the tibial component by ±0.5 mm in the inferior–superior direction, the anterior–posterior and inferior–superior contact forces as well as the anterior–posterior and inferior–superior translations were visualised. To evaluate the varying embedding of the femoral component by ±1° adduction the inferior–superior contact force as well as the abduction–adduction contact moments were investigated.

Unless otherwise specified, 300 load cycles were executed for all force-controlled tests and 40 cycles were executed for the position-controlled tests to analyse the above-mentioned parameters. Thereby, the last five cycles were averaged and analysed.

## 3. Results

### 3.1. Influence of Control Method

**Figure 6:**
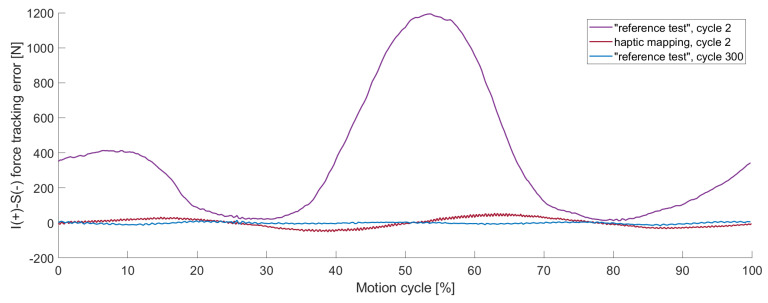
The inferior–superior (I-S) force tracking error of individual cycles, precisely cycle 2 with ILC of the “reference test” (purple), cycle 2 of the haptic mapping prior to the ILC cycles (red) and cycle 300 of the “reference test” (blue). The highest tracking error is found for the 2nd cycle of the “reference test”.

In this preceding test, one cycle of haptic mapping lasted twice as long as the following ILC cycles. The haptic mapping, which was performed prior to the ILC test, showed different results compared to the first ILC cycles. This is visualised for the inferior–superior force in Figure 6 by displaying the tracking error. Whereas the haptic mapping showed a small deviation, the first cycles of the ILC test were overloaded with up to 1200 N.

#### 3.1.1. Comparison of ILC and PI Mode

In Table 2, the RMSE of all six controlled DOF are presented for different cycles to evaluate the performance of the ILC with regard to the tracking errors compared between the ILC and PI controller. For the comparison, five cycles were averaged, resulting in the presentation of cycles 2–7, cycles 96–100, cycles 196–200, cycles 296–300.

The ILC managed to reduce the tracking error consecutively for all six DOF. As it was shown before, the major reduction happened during the first cycles, which was also caused by a huge tracking error occurring during the first cycles of ILC tests. Nevertheless, after 100 cycles the ILC reduced the tracking error by more than 99% for the inferior–superior force and more than 36% of the external–internal rotation moment. Until cycle 200, the tracking error in all DOF was further reduced, except the flexion angle, which had converged earlier. There was a stagnation or a minor increase in the tracking error of four DOF for cycle 300, whereas the two others, namely anterior–posterior force and abduction–adduction moment were still reduced.

**Figure 7:**
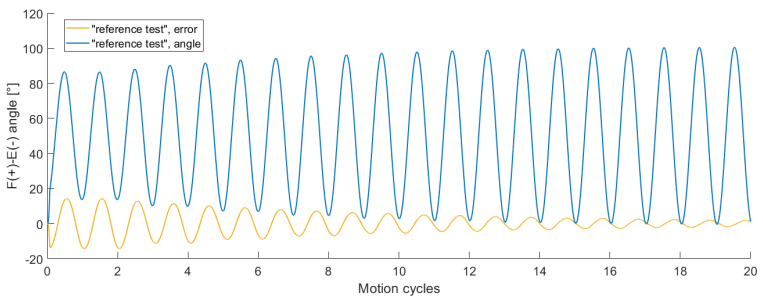
The flexion–extension (F-E) angle (blue) and the tracking error of the flexion–extension angle (yellow) of the first 20 cycles of the “reference test” plotted relative to the number of motion cycles. The tracking error for the F-E angle decreased steadily over the first cycles.

In Figure 7 the flexion angle as well as the tracking error of the flexion angle of the first 20 cycles of the “reference test” are visualised. Most of the reduction in the tracking error occurred in the first few cycles. Whereas the second cycle (the first cycle is neglected because of transition time) had an amplitude of less than 73°, the 20th cycle had an amplitude of roughly 101°. The convergence of the tracking error applies to all DOF, as shown in the previous paragraph.

#### 3.1.2. Occurrence of Oscillation

**Figure 8:**
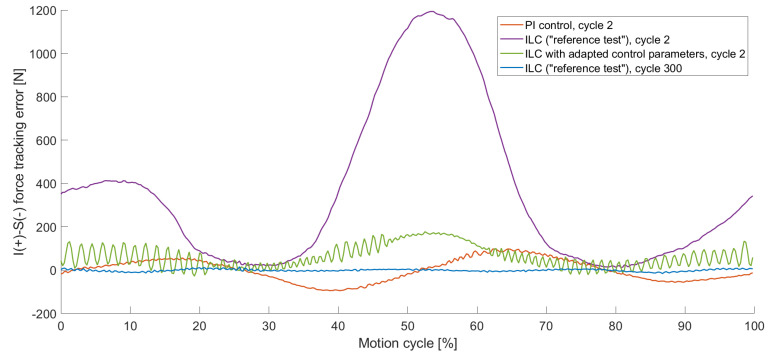
The inferior–superior (I-S) force tracking error for different control parameters of individual load cycles, precisely cycle 2 with PI control (orange), with ILC of the “reference test” (purple) and with ILC with adapted control parameters (green) as well as cycle 300 of the “reference test” (blue). For the 2nd cycle ILC with adapted control parameters, the tracking error was reduced, but oscillations occurred.

**Figure 9:**
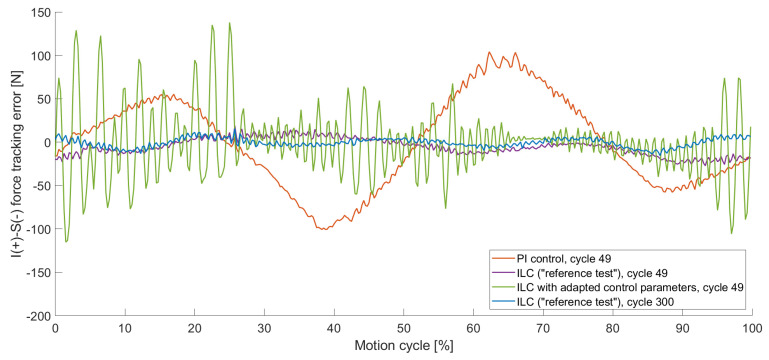
The inferior–superior (I-S) force tracking error for different control parameters of individual load cycles, precisely cycle 49 with PI control (orange), with ILC of the “reference test” (purple) and with ILC with adapted control parameters (green) as well as cycle 300 of the “reference test” (blue). The oscillation within the ILC with adapted control parameters increased with the number of cycles.

During the first load cycles, the results of the ILC varied highly between individual cycles. The second cycle of the different control parameters is visualised in Figure 8. The inferior–superior force that was controlled to zero reached a maximum load of 98 N for the PI control in the second cycle, whereas the ILC with the same control parameters resulted in 1194 N in the second cycle (Figure 8). For manually adapted control parameters (Table A2), the maximum force reduced to 173 N for the ILC in the second cycle. With increased control parameters, an oscillating effect occurred, which was noticeable in early cycles (visualised in Figure 8) but increased in later ones (Figure 9). The oscillation had a high impact on the RMSE for the individual cycles, which are presented in Table 3. For the second cycle, the RMSE of the PI control, as well as the ILC with adapted control parameters, featured a lower RMSE compared to the “reference test”. Nevertheless, in cycle 49, the tracking error of the “reference test” was reduced by 95%, whereas the ILC with the adapted values controlled test showed a tracking error of 354% compared to the “reference test”. The results of the PI control did not differ in a relevant way in between the second and the 49th cycle.

### 3.2. Repeatability and Kinematic Reproducibility

The “reference test” was repeated several days after the initial test to assess the repeatability. To analyse the kinematic reproducibility, the load case was performed purely kinematically with the translational and rotational position output obtained from the “reference test”. To evaluate the resulting contact forces of this kinematic reproducibility, it was necessary to initially verify how accurately the simulator can replicate the joint kinematics. The results in Table 4 show a high precision of the joint simulator with translational and rotational RMSEs of ≤0.01 mm and ≤0.04°, respectively.

**Figure 10:**
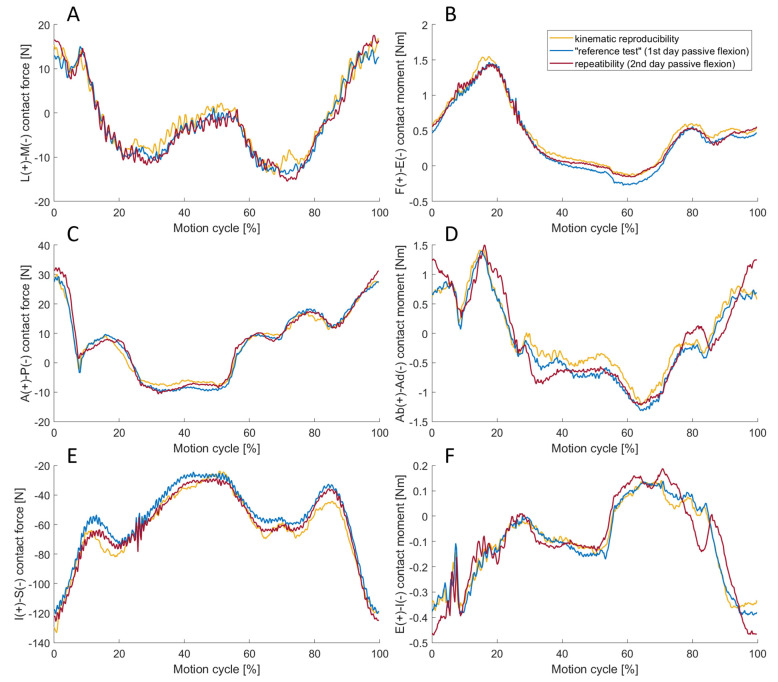
The lateral–medial (L-M) contact force (**A**), the flexion–extension (F-E) contact moment (**B**), the anterior–posterior (A-P) contact force (**C**), the abduction–adduction (Ab-Ad) contact moment (**D**), inferior–superior (I-S) contact force (**E**) and external–internal rotation (E-I) contact moment (**F**) for the kinematic reproducibility (yellow), the repeatability test (red) as well as the “reference test” (blue). The dynamic results of the reference test could be reproduced in both the kinematic reproducibility and the repeatability tests.

The contact forces and moments of the repeatability and reproducibility tests are shown in Figure 10. The contact forces and moments barely differed from the “reference test” for both tests. The lowest correlation was found for the external–internal rotation contact moment of the reproducibility test with a Pearson correlation factor of 0.93, as shown in Table 5.

### 3.3. Effect of the Flexible Bellows

**Figure 11:**
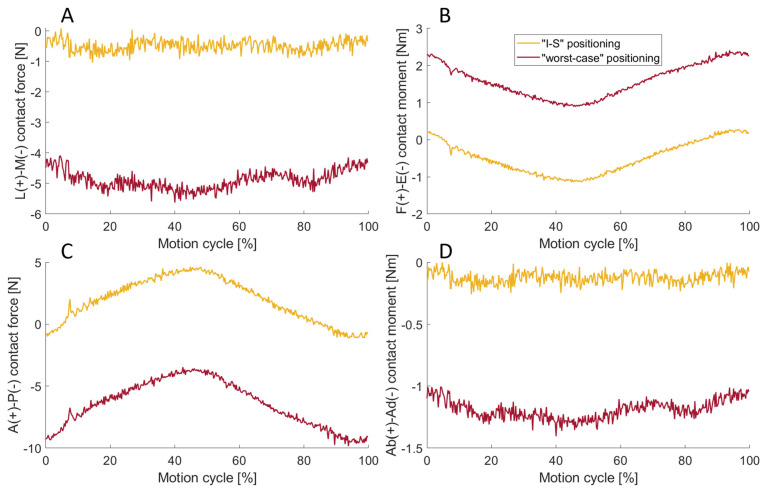
The lateral–medial (L-M) contact force (**A**), the flexion–extension (F-E) contact moment (**B**), the anterior–posterior (A-P) contact force (**C**) and the abduction–adduction (Ab-Ad) contact moment (**D**) for the “I-S” (yellow) as well as for “worst-case” positioning test (red). Both ‘I-S’ and ‘worst case’ positioning had an effect on the measured dynamics.

In Figure 11, the force and moment profiles recorded by the force transducer with the flexible bellows installed are shown in two positions with force set to zero. If the force transducer was set to zero in “I-S” positioning, this mainly affected the measured anterior–posterior contact force and the flexion–extension contact moment with a maximum deviation of 4.68 N and −1.15 Nm, respectively. In the case of the “worst-case” positioning, deviations in the lateral–medial contact force and the abduction–adduction contact moment were also observed. The maximum deviation in the anterior–posterior direction was −9.60 N and the flexion–extension contact moment deviated from the zero line by up to 2.36 Nm.

### 3.4. Effect of the Waveform Frequency

**Figure 12:**
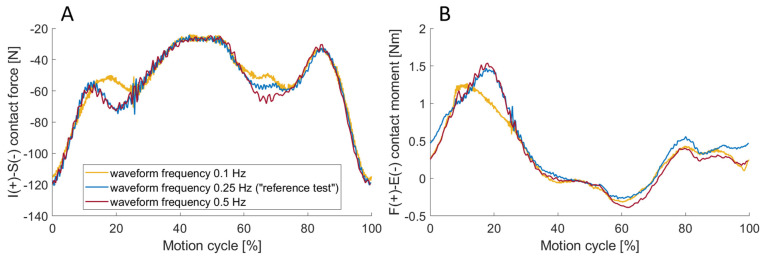
The inferior–superior (I-S) contact force (**A**) and flexion–extension (F-E) contact moment (**B**) for different waveform frequencies of 0.1 Hz (yellow), 0.25 Hz (blue) and 0.5 Hz (red). The maximum flexion moment increased slightly for both 0.25 Hz and 0.5 Hz compared to 0.1 Hz waveform frequency.

All forces and moments measured were very similar for the three waveform frequencies tested, with exemplarily visualisation for the inferior–superior contact force and for the flexion–extension contact moment in Figure 12. The plotted curves are the mean of the cycles 296–300. The maximum vertical load for all three waveform frequencies was between 117 N and 121 N. The maximum flexion moment, the only dynamic variable that is not controlled but was imposed by the rotation, differed slightly between the three waveform frequencies. It slightly increased with the waveform frequency from 1.3 Nm to 1.5 Nm for both 0.25 Hz and 0.5 Hz.

Table 6 shows the RMSE of the six controlled DOF in the case of different waveform frequencies. It was observed that the tracking errors between the different waveform frequencies are minor and without apparent tendency. Among the forces, the tracking error was greatest in the anterior–posterior DOF. RMSEs of the flexion–extension angles were below 0.1°. The tracking error of the abduction–adduction moments and external–internal rotation moments were within a range of 0.2 Nm to 0.3 Nm for all waveform frequencies.

### 3.5. Effect of Lubrication

**Figure 13:**
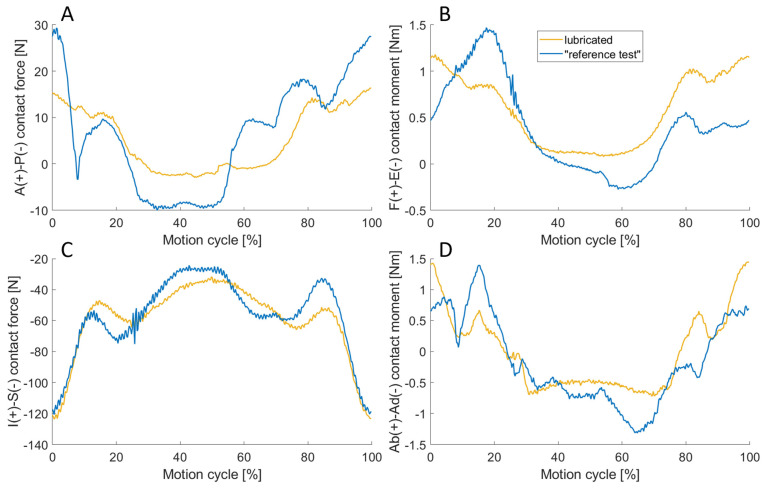
Anterior–posterior (A-P) contact force (**A**), flexion–extension (F-E) contact moments (**B**) inferior–superior (I-S) contact force (**C**) and abduction–adduction (Ab-Ad) contact moments (**D**) under lubricated and non-lubricated (“reference test”) conditions. The symmetry observed in the lubricated test is less apparent for the non-lubricated reference test.

In Figure 13, the forces and moments from the experiment with silicone oil are compared to the “reference test” without lubrication. In direct comparison, it was noticeable that the curves of the lubricated test showed a symmetry with the axis of symmetry at 50% cycle. In contrast, the curves of the non-lubricated reference experiment differed depending on whether the implants were entering or leaving flexion. This applied for all DOF, exemplarily for the anterior–posterior and inferior–superior contact force as well as abduction–adduction contact moment. Additionally, it was particularly evident in the flexion moment. At 18.5% and 81.5% of the cycle, the flexion angle was 30°. In the experiment without lubrication, the measured moment was 1.43 Nm and 0.48 Nm, while the difference in the lubricated experiment was considerably smaller with 0.83 Nm and 0.99 Nm. With respect to the forces, the RMSE of the tracking error was lower in the lubricated experiment, while it remained unchanged for the rotational DOF, as shown in Table 7.

### 3.6. Sensitivity to Embedding of the Implant Components

**Figure 14:**
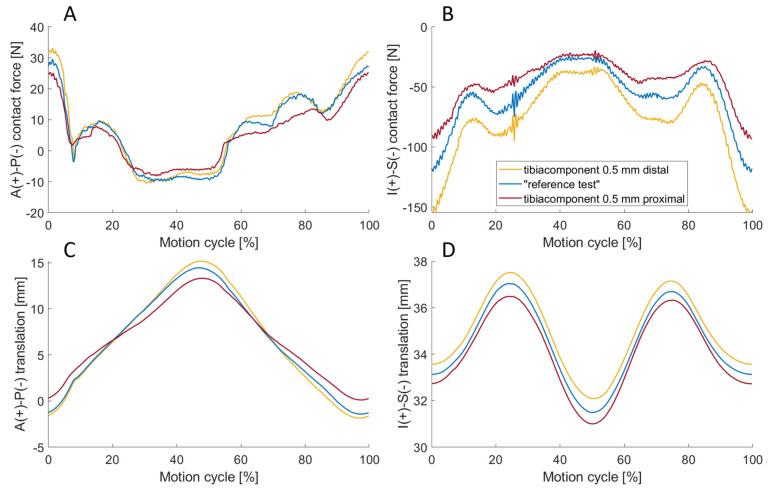
The anterior–posterior (A-P) contact force (**A**), the inferior–superior (I-S) contact force (**B**), the anterior–posterior (A-P) translation (**C**) and the inferior–superior (I-S) translation (**D**) for simulated embedding situations of the tibia component by 0.5 mm distal (yellow) and 0.5 mm proximal (red) and without forced offset from the embedding, which is equivalent to the “reference test” (blue). I-S contact force decreases when shifting the tibial component through simulated embedding situation from distal to proximal.

Shifting the tibial coordinate system by ±0.5 mm along the inferior–superior axis by manipulating the reference pose also changed the ligament attachment points relative to the tibia insert, and thus the induced virtual ligament forces. The impact of this adjustment on resultant joint dynamics is shown in Figure 14. Depending on the inferior–superior translation from −0.5 mm to +0.5 mm, the maximum load manifested in extension at approximately 93.2 N, 120.2 N, and 155.6 N, respectively. Accordingly, the absolute anterior–posterior forces were also higher. Considering the anterior–posterior translation, the range of motion increased with increasing inferior–superior translation with approximately 13.18 mm, 15.83 mm, and 16.99 mm.

**Figure 15:**
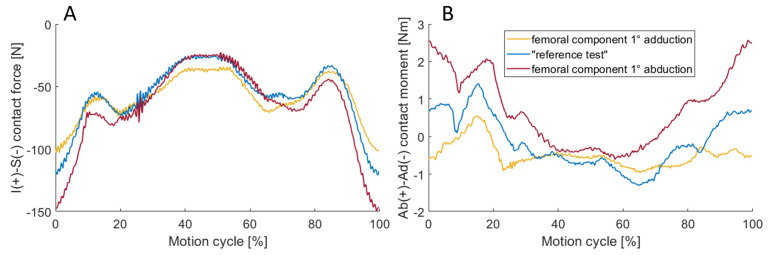
The inferior–superior (S-I) contact force (**A**), the abduction–adduction (Ab-Ad) contact moment (**B**) for simulated embedding situations of the femoral component by 1° adduction (yellow) and 1° abduction (red) and without forced offset from the embedding, which is equivalent to the “reference test” (blue). Ab-Ad contact moment decreases when shifting the tibial component through simulated embedding situation from abduction to adduction.

The inferior–superior contact force and abduction–adduction contact moment for the different abduction reference poses are represented in Figure 15. Raising the abduction angle led to higher forces and absolute maximal abduction moments of 0.95 Nm, 1.39 Nm, and 2.57 Nm for −1°, 0° (reference) and +1° abduction angle.

## 4. Discussion

The aim of our study was to describe the opportunities and limitations of the advanced VIVO^TM^ joint simulators capable of applying realistic joint loading in six DOF. With improving movement and measuring accuracy, the importance of correct placement of the implant components relative to the simulator in six DOF increases. The usage of the specialised convention described by Grood and Suntay [33], instead of established Cartesian Euler angles (Tait–Bryan/Kardan angles) to describe kinematics and dynamics, raises the complexity of preparing input and evaluating output data [36]. Without the invention of advanced control algorithms such as the ILC, proper control for all six DOF would be hardly possible [14]. Additionally, the ability to define virtual ligaments in an efficient way opens new possibilities, as it is still technically difficult to realistically simulate the joint capsule and ligament structures during experiments [37,38,39,40]. But it also causes new challenges mainly concerning implant fixation and control methods of the joint simulator. This study represented the effort to evaluate and describe different test-setup conditions in detail, complemented by insights into the utilisation of the advanced VIVO^TM^ joint simulator.

The design of the VIVO^TM^ joint simulator allows great flexibility regarding the joint situation and load cases to be investigated. The simulator only dictates certain positioning and orientations of the two coordinate systems of the joint pairing. In the present study, the functionality of the VIVO^TM^ joint simulator is demonstrated for biomechanical testing of total knee endoprostheses, for which a certain integration into the simulator is suggested by the manufacturer and is also plausible due to one main DOF—the flexion–extension motion. Thereby, the VIVO^TM^ joint simulator is able to concentrate high ranges of motion and loads around all six DOF in a rather small workspace (compared to test setups using industrial robots). For joints with a larger range of motion, such as the shoulder, users must consider in advance how to integrate the joint pairing to replicate the load case to be applied.

### 4.1. Influence of Control Method

The haptic mapping process describes cycles that are run prior to the actual testing cycles to improve control performance during these tests. Comparing the haptic mapping cycles with the ILC cycles that ran immediately afterwards, the haptic mapping provided better results in terms of tracking errors, which becomes relevant when the load limits of the simulators are reached. During our test with an expected load of 130 N, 1250 N and therefore 25% of the load limitation of the VIVO^TM^ joint simulator [15] was reached during the second cycle of the ILC test. This tracking error did not occur for tests performed with a PI control. In our tests, the ILC started to deliver better results when it came to reducing the tracking error in less than 20 cycles compared to the PI control. The assumption that overloading can be prevented by increasing the control parameters could be confirmed with this load case. In this study, we could show that increased control parameters, however, led to the problem of an oscillating effect which intensified over further cycles.

The concept of ILC has been known for more than 50 years [41] and the implementation into a joint simulator delivered fully automated results that could not have been achieved by manual PI parameterisation. With the additional usage of virtual ligaments, the control capabilities of the simulator were influenced in a way that the experiment began to oscillate for the same control values that could be used problem free without active virtual ligaments. This led to the difficulty that experiments performed with the default control parameters must be cancelled to avoid overloads in the first cycles, while increasing the control parameters led to an oscillating effect that can progressively worsen using the ILC. We found that changing the control parameters during the runtime of the experiment (e.g., starting the experiment with custom control parameters and lowering them after cycle 10 to default parameters) can circumvent this problem.

### 4.2. Repeatability and Kinematic Reproducibility

The reproducibility of the results of the reference load case could be confirmed by repeating it on a second day of testing, as the overall correlation is considerably large. The DOF with the lowest correlation was the external–internal rotation contact moment, which in this setup is therefore considered the most sensitive DOF. This can be explained by the fact that the external–internal rotation is almost geometrically unconstrained, and the acting moments were very low in the range of −0.5 Nm to 0.2 Nm. To test the purely kinematic repeatability, all six DOF were position or rotation controlled. This load case required a very high degree of accuracy, as even small deviations could have resulted in high contact forces when the joint partners collided. In fact, these concerns were unwarranted, given the correlation factors of more than 0.98 for the dynamic variables. The correlation (apart from the inferior–superior contact force) turned out to be even higher than in the repeatability test. However, it must be noted that the kinematic reproducibility test took place on the same day as the “reference test”, and if conducted on a different day (with a new determination of the reference pose), it might result in a lower correlation. In summary, it can be concluded that the VIVO^TM^ joint simulator is capable of accurately replicating a previously executed dynamic load case with high precision in terms of kinematics.

### 4.3. Effect of the Flexible Bellows

To investigate the influence of the flexible bellows on force measurement, the force transducer was set to zero at two different positions of the lower actuator and the kinematics of the previous test were performed. In the test where the transducer was set to zero as the lower actuator was moved down 10 mm (“I-S” positioning), an anterior–posterior force of −1.1 to 4.7 N was measured during the test. The lateral–medial forces and the abduction–adduction moment remained constant at nearly zero. This can be explained by the fact that the translational range of motion is greatest in the anterior–posterior direction (more than 15 mm) and minimal in the lateral–medial direction (1.1 mm). Accordingly, the flexible bellows exert greater tensile forces in the anterior–posterior direction, resulting in an increase not only in the measured anterior–posterior force but also in the flexion–extension moment. In the second position, the “worst-case” positioning effects on the lateral–medial force and the abduction–adduction moment are also apparent. This is caused by the flexible bellows exerting more tension in the lateral–medial and anterior–posterior directions in the zero position of the load cell than in the reference pose. Comparing the curves of both positions, it is evident that they run almost in parallel and are only separated by a constant offset. As the lower actuator precisely follows the same trajectory in both experiments, it can be deduced that the tensile forces of the flexible bellows increase linearly with displacement, indicative of a constant material stiffness of the flexible bellows.

The maximum flexion–extension moment caused by the flexible bellow (“I-S” positioning) was 1.1 Nm, already accounting for approximately 78% of the maximum moment in the “reference test”. In general, or at least if the expected moments are small, we recommend not using the flexible bellows and relying on alternative protective devices when working with substances such as serum in order not to hamper the experiments.

### 4.4. Effect of the Waveform Frequency

When comparing the different waveform frequencies, no clear dependencies could be identified regarding either the measured forces or the tracking error. The assumption that different angular velocities would have an impact on the friction behaviour and, consequently, on the dynamics of the system, could not be confirmed within this load case. The comparison of the tracking error also showed that a higher waveform frequency did not negatively affect the control. Therefore, future investigations can be conducted at higher frequencies to reach the defined cycle count more quickly without the concern of increasing the tracking error.

### 4.5. Effect of Lubrication

A broad variety of approaches for the lubrication of experimental investigations in joint endoprostheses is reported in the literature [3,4,14,21,25,26,27,28,29,30]. Due to the effective self-lubricating properties of polyethylene, additional lubrication is often dispensed within experimental investigations, i.e., studies with test rigs [25], robots [14] and wear simulators [26]. The standard medium for wear testing of joint endoprostheses is bovine serum, according to ISO 14242 [3] or 14243 [4]. However, alternative lubricants have been developed to mimic the characteristic behaviour of human synovia [27,28,29]. In addition, some studies used silicone oil as a lubricant to enforce ideal lubrication behaviour [21]. Herrman et al. [30] used deionised water for joint lubrication in a robot-based approach for the dynamic testing of total hip replacements. This enabled a reduction in the absolute value of friction torque over the whole motion cycle compared to the dry test.

The results of our present study indicate that lubrication affects test results. Through lubrication, the joint simulator could control the load case more precisely, as evidenced by the lower tracking errors in forces and the symmetry of the measured curves. This could be attributable to the minimisation of friction through lubrication. For example, in order to keep the anterior–posterior force constant at 0 N, the simulator attempts to create a motion by equating the contact and friction forces measured by the force transducer with the virtually calculated ligament forces. This is likely easier to control due to the elimination or reduction in friction forces in the lubricated experiment.

Because not only the friction forces but also the tracking errors differed between the lubricated and non-lubricated experiments, the actual influence of lubrication on the joint dynamics is challenging to quantify.

### 4.6. Sensitivity to Embedding of the Implant Components

Regarding the simulated implant embedding, it was shown that even small deviations of ±0.5 mm inferior–superior tibial shift and ±1° femoral abduction–adduction have a major influence on the resulting joint dynamics. In the case of the inferior–superior shift, the maximum inferior–superior load was approximately 22% lower (−0.5 mm shift) and 29% higher (+0.5 mm shift). With the higher ligament forces, the range of motion of the implant in the anterior–posterior direction increased. This occurred due to the amplified effect of the virtual ligaments on the resulting motion. For example, the PCL pulls the tibia further anteriorly while in flexion [42]. Tilting the femoral abduction axis also influenced the maximum inferior–superior force. The fact that the inferior–superior forces increased with the angle of abduction suggests that the medial side of the ligamentous apparatus was stiffer than the lateral side. This effect was further enhanced by the simulated rotation of the tibial coordinate system (+1° abduction). This could also be seen in the abduction moment, which was more pronounced at higher abduction angles. However, when the tibial coordinate system was rotated by −1° abduction, the absolute moment about the anterior–posterior axis decreased during the test, indicating that the ligamentous apparatus was more balanced compared to the “reference test”.

### 4.7. Limitations

In our present study, several limitations must be taken into account. First of all, it must be noted that only one reference load case and only one implant design was analysed. It cannot be ruled out that some of the results might differ if another load case or implant had been considered. For instance, the measured loads were low compared to walking [4,35]. Differences in contact forces caused by the ligaments during embedding, for example, might have had a considerably smaller influence on the resulting kinematics during walking. However, the load case “passive flexion”, established in the literature [14,43], was selected because it allowed for a neutral evaluation of the implant kinematics on the one hand and focused on the use of the virtual ligament apparatus, which is advanced for simulators, on the other. Furthermore, it was for these reasons that the selection of the implant leaned towards an unconstrained knee design. The PFC Sigma knee system, in particular, is an unloaded bicondylar posterior cruciate ligament-retaining knee design, that was used in previous studies to evaluate joint dynamics [44,45]. The ligament apparatus used in this study was adapted from a previously conducted musculoskeletal multibody study on a total knee endoprosthesis [34], and therefore, no known pathology, e.g., genu varum or genu valgus, was considered during the parameterisation of the ligaments. In the future, variations of the ligament apparatus should also be investigated to analyse the behaviour of the knee endoprosthesis under these conditions. With regard to the control method, it should be noted that the parameters of the PI control were optimised by trial and error. Strategies and empirical formulas such as those used by Ziegler and Nicholas [46] were not applied. On the other hand, due to the large number of tests, the cycles for a test were limited to a maximum of 300, although the ILC should theoretically be able to further minimise the tracking error even beyond this number. The reason why no influence of the waveform frequencies on the measured forces could be determined in this study, is possibly due to the fact that these tests were carried out in a non-lubricated condition. However, for hydrodynamic tests a variable friction force behaviour can be described by the Stribeck curve [47]. Investigations of different waveform frequencies in the lubricated condition should therefore also be analysed in future.

Furthermore, this study has demonstrated that even minor changes of the implant embedding situation considerably affect ligament forces; therefore, variations due to deformations or inherent bearing play in the actuators could similarly influence the load case through imprecisely determined ligament attachment points. In general, the accuracy of the relative kinematics between the upper and lower actuator recorded by the VIVO^TM^ joint simulator may be measured in future studies using an additional external measuring system. The VIVO^TM^ joint simulator always localises the femoral coordinate system at the intersection of the axes of the flexion and abduction arm. However, it can be observed that the upper structure yields slightly under higher loads, which would result in inaccurately recorded relative kinematics.

## 5. Conclusions

The VIVO^TM^ joint simulator, as an example of an advanced joint simulator, offers a broad diversity of features that are useful to test joint endoprostheses under dynamic load scenarios. Nevertheless, the handling of the simulator is influenced by different parameters, which has been analysed in this study. Based on the different test parameters in the passive flexion–extension load case, the following insights were obtained: (1) the control algorithm ILC was able to considerably reduce tracking errors with ease compared to the PI control; (2) the frequency of the waveform had no apparent influence on the results; (3) when using the virtual ligaments, minor embedding errors had greater impact; (4) the investigated load case could be kinematically reproduced with high accuracy; (5) flexible bellows designed to protect the simulator from impurities might hamper the test results; (6) lubrication affected not only the implant dynamics but also the simulator control.

In future investigations, further different load cases and different implant designs will be considered. The present study highlights challenges during the utilisation of advanced joint simulators. This may support scientists and engineers in employing advanced joint simulators for functional and tribological analysis of joint endoprostheses.

## Figures and Tables

**Table 1:** Implemented ligament parameters (stiffness and reference strain) considered for testing of the P.F.C.^®^ Sigma knee endoprostheses in the joint simulator. The ligament stiffness is expressed in Newton per unit strain.

Name	Stiffness [N]	Reference Strain [%]
aPCL	3000	−11.373
pPCL	1500	−7.552
aMCL	1500	4.321
pMCL	1500	4.298
dMCL	1500	−2.783
aLCL	1800	5.741
pLCL	2250	3.912
pOPL	1250	5.826
dOPL	1250	5.891
APL	1500	−1.642
lpCAP	2500	−1.167
mpCAP	2500	−2.366

**Table 2:** Root mean square error (RMSE) of PI-controlled cycles 2 to 7 as well as iterative learning control (ILC) cycles 2 to 7, cycles 96 to 100, cycles 196 to 200 and cycles 296 to 300 for the tracking error of the “reference test” for the lateral–medial (L-M), anterior–posterior (A-P), inferior–superior (I-S), flexion–extension (F-E), abduction–adduction (A-A) and external–internal rotation (E-I) directions.

RMSE	L-M Force Tracking Error [N]	A-P Force Tracking Error [N]	I-S Force Tracking Error [N]	F-E Angle Tracking Error [°]	A-A Moment Tracking Error [Nm]	E-I Moment Tracking Error [Nm]
PI cycle 2–7	13.0	67.0	52.3	2.54	1.2	0.6
ILC cycle 2–7	14.0	51.3	196.9	8.26	2.4	0.5
ILC cycle 96–100	2.1	19.9	6.7	0.04	0.6	0.3
ILC cycle 196–200	1.5	13.3	5.2	0.04	0.4	0.3
ILC cycle 296–300	1.6	10.1	5.2	0.04	0.3	0.3

**Table 3:** Root mean square error (RMSE) of the inferior–superior (I-S) tracking error of individual load cycles, precisely cycle 2 with PI control, with ILC of the “reference test” and with ILC with adapted control parameters as well as cycle 300 of the “reference test”.

RMSE of the I-S Tracking Error [N]	Cycle 2	Cycle 49	Cycle 300
ILC “reference test”	506.5	11.1	5.2
ILC adapted values	81.2	39.3	-
Pi control	51.6	52.2	-

**Table 4:** Root mean square error (RMSE) of the kinematic reproducibility for the lateral–medial (L-M), anterior–posterior (A-P), inferior–superior (I-S), flexion–extension (F-E), abduction–adduction (A-A) and external–internal rotation (E-I) directions.

RMSE of the Tracking Error	L-M Translation Tracking Error [mm]	A-P Translation Tracking Error [mm]	I-S Translation Tracking Error [mm]	F-E Angle Tracking Error [°]	A-A Angle Tracking Error [°]	E-I Angle Tracking Error [°]
	0.01	0.01	0.00	0.04	0.04	0.01

**Table 5:** Correlation factors of the contact forces and moments concerning the repeatability and kinematic reproducibility test with respect to the reference test for the lateral–medial (L-M), anterior–posterior (A-P), inferior–superior (I-S), flexion–extension (F-E), abduction–adduction (A-A) and external–internal rotation (E-I) directions.

Correlation Factor	L-M Contact Force	A-P Contact Force	I-S Contact Force	F-E Contact Moment	A-A Contact Moment	E-I Contact Moment
Repeatability	0.98	0.99	1.00	1.00	0.96	0.93
kinematic reproducibility	0.99	0.99	0.98	1.00	0.99	0.99

**Table 6:** Root mean square error (RMSE) of the tracking error for three different waveform frequencies (“reference test” was conducted with 0.25 Hz) for the lateral–medial (L-M), anterior–posterior (A-P), inferior–superior (I-S), flexion–extension (F-E), abduction–adduction (A-A) and external–internal rotation (E-I) directions.

RMSE of the Tracking Error	L-M Force Tracking Error [N]	A-P Force Tracking Error [N]	I-S Force Tracking Error [N]	F-E Angle Tracking Error [°]	A-A Moment Tracking Error [Nm]	E-I Moment Tracking Error [Nm]
f = 0.1	1.7	9.8	3.9	0.01	0.3	0.2
f = 0.25	1.5	10.1	5.1	0.04	0.3	0.3
f = 0.5	1.4	10.6	5.1	0.04	0.2	0.2

**Table 7:** Root mean square error (RMSE) of the tracking error of all degrees of freedom for the lubricated and non-lubricated “reference test” for the lateral–medial (L-M), anterior–posterior (A-P), inferior–superior (I-S), flexion–extension (F-E), abduction–adduction (A-A) and external–internal rotation (E-I) directions.

RMSE of the Tracking Error	M-L Force Tracking Error [N]	A-P Force Tracking Error [N]	S-I Force Tracking Error [N]	F-E Angle Tracking Error [°]	A-A Moment Tracking Error [Nm]	I-E Moment Tracking Error [Nm]
lubricated	0.9	6.6	4.0	0.03	0.3	0.3
unlubricated (“reference test”)	1.5	10.1	5.1	0.04	0.3	0.3

## Data Availability

Data are contained within the article. Raw data can be shared by the authors on request.

## Appendix A

**Table A1:** Control parameters of the “reference test”.

	Force			Displacement	
Control Parameters	Integral Gain (mm/s)/N	Proportional Gain mm/N	Maximum Rate (mm/s)	Integral Gain (mm/s)/mm	Servo Bandwidth
ML	0.02	0.003	10	5	6
AP	0.02	0.003	10	5	6
VL	0.1	0.005	10	5	6
			(deg/s)		
FL	10	0.2	0.2	20	5
Abd	10	0.2	0.2	20	6
IE	10	0.2	0.2	20	6

**Table A2:** Adjusted control parameters.

	Force			Displacement	
Control Parameters	Integral Gain (mm/s)/N	Proportional Gain mm/N	Maximum Rate (mm/s)	Integral Gain (mm/s)/mm	Servo Bandwidth
ML	0.02	0.003	10	5	6
AP	0.02	0.003	10	5	6
VL	0.1	0.02	15	5	6
			(deg/s)		
FL	10	0.2	0.2	20	5
Abd	10	0.2	0.2	20	6
IE	10	0.2	0.2	20	6
